# Mapping common job demands and job resources for large-scale community health worker programmes in Southern Africa: protocol for a scoping review

**DOI:** 10.1136/bmjopen-2026-116412

**Published:** 2026-05-29

**Authors:** Rachel S Coley, Linda Shuro, Renier Coetzee, Hanani Tabana

**Affiliations:** 1 University of the Western Cape, Cape Town, South Africa; 2 SAMRC Health Systems to Services Research Unit, University of the Western Cape, Cape Town, South Africa

**Keywords:** Occupational Stress, Primary Health Care, Health Workforce, Africa South of the Sahara

## Abstract

**Abstract:**

**Introduction:**

High burdens of infectious and non-communicable diseases and human resource shortages strain primary healthcare systems in Southern Africa. Despite the widespread use of community health workers (CHWs) across the region, the existing literature offers no holistic exploration of the characteristics of CHWs’ work. The Job Demands-Resources model provides a framework to enable such a review, positing that two aspects of working conditions—job demands and job resources—are unique to each workplace but have universal impacts on workers’ health, motivation and the achievement of organisational aims.

**Methods and analysis:**

The population, concept and context framework will guide the development of a comprehensive search strategy to source reports on CHWs working in large-scale programmes that explore job demands and job resources within at least one Southern African Development Community country. Searches to identify peer-reviewed articles and grey literature will be done in CHW Central, CINAHL, PubMed, Scopus and the WHO Library with citations purposively mined. Eligibility will be limited to reports published in English after the Declaration of Ouagadougou on Primary Health Care in 2008, as this ushered in the modern era of large-scale CHW programmes in Africa. Following publication of this protocol, two reviewers will independently complete screening and full-text review, with the lead author driving data extraction and coding. The results will include organising job demands and job resources into themes, documenting the types of research that articulate these themes and identifying potential knowledge gaps. The review’s methodology and the inclusion of various types of literature will support replicability and comprehensiveness.

**Ethics and dissemination:**

Formal ethical approval is not required. Results will take the form of a summative article, and additional publications may be considered if the results warrant more in-depth reporting. The evidence from this review may also inform the creation or improvement of programmes.

**Trial registration number:**

INPLASY202580052.

**STRENGTHS AND LIMITATIONS OF THIS STUDY**

- This protocol describes a novel approach to reviewing occupational characteristics for community health workers, grounded in the Job Demands-Resources model.
- The protocol’s methodology aligns with the Preferred Reporting Items for Systematic Reviews and Meta-Analyses extension for Scoping Reviews and Arksey and O’Malley’s scoping review framework, both of which will guide the approach to enhance comprehensiveness and replicability.
- The focus on the broad characteristics of job demands and job resources across the Southern African Development Community allows for mapping a variety of realities for community health workers in large-scale programmes and comparison between country programmes but limits the ability to focus on nuances within an individual programme or demand/resource.
- The use of five databases, including those with grey literature, as well as the purposive selection of included article citations and those from evidence synthesis research identified through the search strategy, will enable comprehensive mapping of the available evidence.
- The exclusion of any non-English articles may exclude some evidence from non-English-speaking countries.

## Introduction

 The 1978 Declaration of Alma-Ata affirms that ‘complete physical, mental and social well-being’ is a fundamental human right.^1^ It establishes primary healthcare (PHC) as a global priority to improve health and development, affirming that communities and individuals must have ownership over their healthcare to meet community health needs.^1^ More recently, the Sustainable Development Goals also underscored that community health workers (CHWs), who are a cadre of health workers with limited formal training but strong connections and understanding of the communities they serve, are a critical component of achieving universal health coverage.^2 3^

National governments and non-governmental programmes have adopted various models for PHC and CHW programmes in the nearly 50 years since Alma-Ata.^4^ Countries and CHW programmes have also been influenced by donor-funded, disease-specific models, often focusing on HIV and tuberculosis (TB).^25^,^7^ Over the past two decades, many countries in Southern Africa have created, revamped or considered programmes with a horizontal, integrated approach to community-based services that often still address infectious diseases but also integrate other strategic priorities like maternal, newborn and child health and non-communicable diseases.^8^,^10^ Programmes of this type are managed by government health authorities and commonly referred to as ‘large-scale’ as they serve broader needs and typically more communities.^511^,^13^ In Southern Africa, large-scale CHW programmes are fully integrated into government PHC systems in the Democratic Republic of Congo, Malawi, Mozambique, Tanzania and Zambia, and on the path to full integration in South Africa and Zimbabwe.^10^

CHWs play diverse roles and often function as intermediaries between their communities and the formal health system. Bringing health information and services to communities also presents challenges in governance and management, as programmes typically have varying levels of integration into the formal health system and involve a wide network of stakeholders, including non-government actors.^14 15^ CHWs implement a wide range of responsibilities in complex and sometimes dangerous environments, placing increasing demands on them that are often not balanced with the training and supervisory resources required to succeed.^1416^,^19^ Research shows that opportunities for personal growth and interpersonal relationships, which are types of job resources, are key drivers of CHW motivation, perhaps even more than financial remuneration.^14 20 21^

Despite enthusiasm for the potential contribution of CHWs to partially address global human resources for health shortages,^22^ there are gaps in what is known about the nature of their work as part of large-scale CHW programmes. This is an especially critical area of exploration given the emphasis on PHC and universal health coverage in the Sustainable Development Goals^23^ and recent reductions in donor assistance, which will further limit vertical CHW programmes.^24^

This review will provide a map of what is known about the nature of CHW work, specifically job demands and job resources, defined in the following section, as part of large-scale CHW programmes in Southern Africa. As it aims to explore the nature of the evidence that exists, potential themes within and across countries, and evidence gaps, a scoping review rather than a systematic review or other evidence synthesis activity is most appropriate.^25 26^ An evidence-mapping approach to data synthesis and dissemination will help stakeholders absorb the large volume of expected information in the final review.^27 28^

This review will serve as evidence for current and future CHW programmes on what is known about these concepts, inform the development of improved models, and assist in the testing of the Job Demands-Resources (JD-R) model for this population.

### Conceptual framework: JD-R model

The JD-R model^29 30^ posits that the characteristics of all occupations can be categorised into job demands and job resources. Job demands refer to aspects of a job that require sustained effort and incur a physiological and/or psychosocial cost. These can include elements of the work environment that are draining, such as social interactions, emotional burden and pressure. Job resources encompass physical, psychological and organisational aspects that facilitate achieving work goals, mitigate the negative impacts of job demands and promote personal growth. Resources can include remuneration, incentives, supportive supervision, equipment and supplies as well as opportunities for growth and advancement.^31^

**Figure 1:**
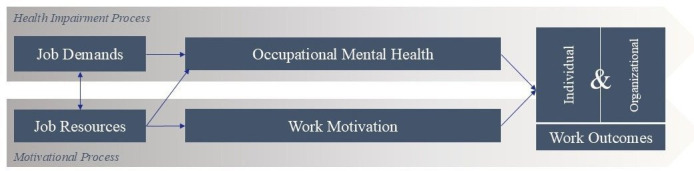
JD-R model. Adapted from Demerouti and Bakker.^31^ JD-R, Job Demands-Resources.

The JD-R model, depicted in figure 1, focuses on the interplay between two processes: a health impairment process, in which job demands lead to stress, burnout and subsequent ill health, which then negatively impact the organisation. The second process is a motivational one, wherein job resources enhance engagement and other aspects of work motivation, while also mitigating the health impairment process and bolstering organisational outcomes.^32 33^ Although the nature of work is unique to each profession, context and the people involved, this interplay is universal.^32 33^

Until now, efforts to understand CHWs’ well-being have focused primarily on motivation and job satisfaction,^21 34 35^ often with a limited focus on potential occupational drivers. Other efforts to explore CHW programmes have relatively narrow research questions focused on supervision,^17 36 37^ knowledge,^38 39^ roles and responsibilities,^18 40 41^ policy,^15 42^ etc. These typically report on a limited set of job demands and job resources under different categorisations, such as strengths and barriers. Evaluations are often the most comprehensive but tend to focus more on programme outputs and impact than on CHWs’ well-being.^43^,^45^

Since it was first outlined in 2001, researchers from around the globe, including Southern Africa,^46^,^50^ have used the JD-R as a theoretical framework to understand occupational mental well-being and explore the impact of job demands and resources on burnout and engagement. The JD-R model has also been expanded to look at theorised antecedents and impacts of burnout and engagement, including depression,^51^ secondary trauma stress,^52 53^ retention,^46^ absenteeism and presenteeism^32^ and work performance.^54 55^ In this research, the JD-R model provides a theoretical framework for the interplay among the characteristics of CHW work. Once documented and organised to align with the model’s foundational components (job demands and job resources), further testing of the model for CHWs in Southern African Development Community (SADC) countries can be conducted. Such research efforts are already planned as part of the lead author’s PhD.

## Methods and analysis

This protocol was developed in accordance with the five-step scoping methodology framework articulated by Arksey and O’Malley.^56^ This framework notes five key steps to a scoping review, which will be used in turn as the review progresses: (1) identifying a research question, (2) identifying relevant studies, (3) selecting studies, (4) charting the data, (5) collecting, summarising and reporting the results. This protocol describes how these steps will be completed, as well as a pilot that was completed in August 2025 to confirm the approach.

The protocol and the resulting study use the JD-R model as the conceptual framework that informs the study’s research questions, as well as the Population, Content and Concept Framework (PCC Framework), which guides the search strategy and development of inclusion and exclusion criteria.

### Research question

The primary and secondary research questions are structured based on the PCC Framework,^26 57^ with details provided in table 1 and through component-specific discussions below. This study’s core research question is: What is known about the job demands and job resources of CHWs working in large-scale programmes in Southern Africa? Secondary questions include: (1) what are the common themes in job demands and job resources within and across country contexts; (2) what kinds of research explore job demands and job resources and (3) what are potential gaps in existing knowledge on job demands and job resources for CHWs in Southern Africa.

**Table 1:** PCC framework for this scoping review and search strategy

Framework component	Study-specific details	Key resources to develop the search strategy
Population	Community health workers (CHWs) whose position is government-managed and focused on primary healthcare (large-scale programme)	Hodgins *et al*’s article on a new taxonomy for CHW programmes as well as country-specific resources on CHW programmes.^1013 45 80^,^82^
Concept	Job demands and job resources^29^	Schaufeli, one of the original authors of the JD-R model, and Taris provide the most comprehensive list of potential job demands and resources known to the researchers.^83^ As this list is not specific to CHWs, it will be supplemented by a review of literature on CHW programmes to customise and improve completeness.^714^,^22 84^
Context	Southern Africa Development Community countries: Angola, Botswana, Comoros, Democratic Republic of Congo, eSwatini, Lesotho, Madagascar, Malawi, Mauritius, Mozambique, Namibia, Seychelles, South Africa, United Republic of Tanzania, Zambia and Zimbabwe^85^	WHO resource for searching literature relevant to lower- and middle-income countries.^86^ The filters, which were developed for use on a country-by-country basis, will be simplified to cover only country names, previous country names (eg, Swaziland for eSwatini) and affiliations.

JD-R, Job Demands-Resources.

### Eligibility criteria

#### Population

Globally, CHWs have a wide range of responsibilities, varying administrative structures and diverse impacts on their communities.^1058^,^60^ This review focuses on CHWs that align with Olaniran *et al*’s broad conceptualisation of the role: ‘Paraprofessionals or lay individuals with an in-depth understanding of the community culture and language, who have received standardised job-related training of a shorter duration than health professionals, and their primary goal is to provide culturally appropriate health services to the community’.^61^ It further limits studies to those that focus on CHWs in large-scale programmes. By focusing on large-scale programmes, this study aims to capture evidence relevant to an integrated organisational structure, wherein CHWs are formalised or are in the process of formalising into the broader health system, and associated challenges and successes of those efforts as they relate to the CHW workplace.

#### Content

This review aims to identify job-demand and job-resource themes, rather than the tasks or responsibilities CHWs must perform within the health system. The latter has already been the focus of several evidence synthesis efforts.^5 18 62^ This review also builds on efforts to understand a specific demand or resource among CHWs and other professions on a broader geographic scale, for example, leadership,^63^ supervision^17^ or workload^64^ through the consolidation of knowledge on a more comprehensive set of workplace conditions within a specific region (Southern Africa). It is theorised that, despite heterogeneity, CHWs likely face shared demands and resources at work, and that a multicountry exploration can identify common characteristics among and between CHW programmes as well as context-specific differences.

Reports focused on CHWs only working on one or two specific disease verticals, like HIV or TB, will be excluded. Reports evaluating specific interventions funded by research entities and nonprofit organisations, even when integrated with large-scale CHW programmes, will be excluded when that support impacted working conditions, for example, through the provision of extra training or equipment.

#### Context

The review will focus specifically on SADC member states. Although SADC is primarily an economic group, it has been used previously as a context delineator for regional health reviews.^65^,^69^ Member countries have nine of the top 10 highest rates of HIV infection in the world,^70^ six of the top 20 countries in TB and three of the top 20 in malaria,^71^ burdening their PHC systems.^72^ High maternal and neonatal mortality and increasing non-communicable disease risks, coupled with human resource shortages, further strain health systems.^71^ Several countries are also plagued by extraordinary rates of violence, homicide and suicide^71^ that impact how people engage with their communities. These dynamics make the region unique and underscore the critical function of CHWs in SADC member states.

### Types of literature

To provide a holistic view, peer-reviewed journal articles and grey literature that meet all the relevant criteria will be included. All reports that report on primary data, regardless of rigour or quality, will be included, except conference papers, dissertations, opinion pieces and protocols. Research focused on evidence synthesis, including policy, systematic and scoping reviews will be excluded; however, these reports will be combined with those that meet all inclusion criteria for the purposive identification of relevant citations for screening.

### Information sources

Five databases were selected for electronic searching: CHW Central, the Cumulative Index to Nursing and Allied Health Literature (CINAHL), PubMed, Scopus and the WHO Library. To identify literature that may have been missed due to the limited number of databases searched and to find relevant grey literature, RSC will review the bibliographies of all included reports for potentially relevant studies that will then go through the study record review process described below.

### Search strategy

By design, this scoping review aims to explore a broad range of workplace characteristics for a population (CHWs) known by various terms, implemented under diverse management structures across numerous countries. This requires a complex search strategy informed by existing research and developed in consultation with experts. Table 1 above provides an overview of key resources to be leveraged in developing a comprehensive search string that reflects the intricacies of this research.

To minimise the inclusion of studies that mention common words or phrases but are not focused on them, terms will only be searched in titles and abstracts. Online supplemental information 1 presents the proposed list of terms to be used in the PubMed search. A similar approach will be used for CINAHL and Scopus. The CHW Central resource library and WHO Library will be searched using their keyword functions to cover the topics explored.

#### Language and date restrictions

All reports published after the signing of the WHO’s Ouagadougou Declaration on PHC,^73^ on 30 April 2008, will be reviewed. This timeframe covers two recent waves of large-scale CHW programme creation or reformulation. The first wave, which came in the years that followed the Ouagadougou Declaration, was characterised by the creation or reinvigoration of programmes in Mozambique, South Africa and Zimbabwe.^13^ The second wave, which began in 2015, aligns with increasing global attention to domestically resourced CHW programmes, including the launch of the Sustainable Development Goals and their emphasis on CHWs and universal health coverage. It also aligns with the founding of the Financing Alliance for Health, which works with African governments to strengthen domestic resources for community health programmes,^20^ and calls to expand CHW programming made by UNAIDS in 2016^74^ and the WHO in 2017 and 2020.^4 75^ This second wave saw the launch of a programme in Tanzania, as well as the introduction of new or reimagined programmes in Madagascar and Malawi.^13^

Due to resource constraints, only peer-reviewed articles and grey literature published in English will be included.

To determine if the planned search approach described was appropriate and would provide sufficient evidence for a review, database searches were piloted on 12 August 2025, as described in table 2, identifying 5242 studies without deduplication.

**Table 2:** Piloted search results from databases conducted on 12 August 2025

	CHW Central	CINAHL	PubMed	SCOPUS	WHO Library
Publications retrieved	346	527	1069	1624	1676

CHW, community health worker.

Database searching will be completed on publication of this protocol.

### Study records

#### Data management and selection process

**Figure 2:**
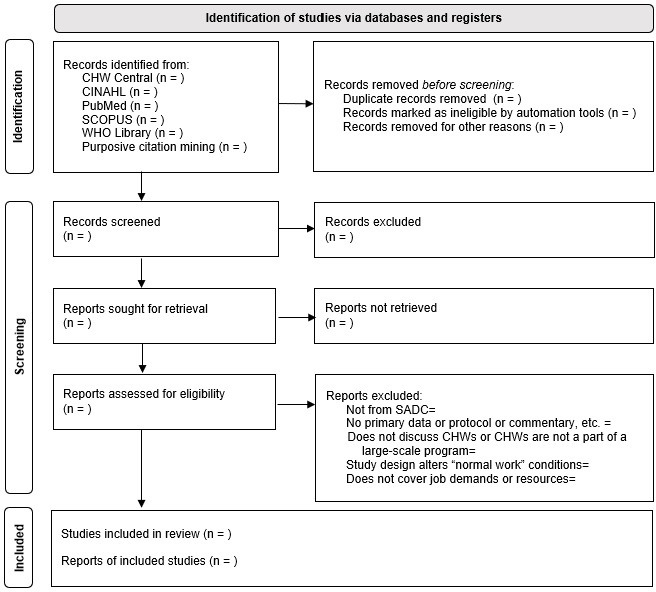
Blank PRISMA flow chart based on the planned approach.^78^ CHW, community health worker; PRISMA, Preferred Reporting Items for Systematic Review and Meta-analysis; SADC, Southern African Development Community.

During the identification phase, initial searches and data will be exported and housed in Papers by ReadCube,^76^ a reference management software system. Search results will be uploaded into Covidence,^77^ a web-based platform for managing systematic and scoping reviews, for deduplication and reviewed against inclusion and exclusion criteria in two phases: title and abstract screening and full-text review. A Preferred Reporting Items for Systematic Reviews and Meta-analyses (PRISMA) flow chart,^78^ customised for this review, will present the results of these processes, as shown in figure 2. Two independent reviewers (RSC and LS) will screen abstracts and full articles, with a third reviewer (HT) resolving any disputes.

To test this process, a random 20% of the results from the PubMed pilot (214 studies) went through the identification process described in the PRISMA flow chart. Reviewers had discussions throughout the process to clarify decision criteria and work processes. Piloting revealed that determining whether to include or exclude a study based on the population was challenging, given the unique management and financial structures in each country or subregion. To facilitate consistent reviews, decision algorithms were developed for screening (online supplemental information 2) and full-text reviews (online supplemental information 3).

#### Data collection

A Covidence data extraction template will be customised to align with review objectives. RSC will extract (see table 3) qualitative and quantitative data from all included studies, including both grey and peer-reviewed literature, to organise study content on job demands and job resources into categories (listed in table 3) and record study information, geographic focus, methodology and CHW design information. Further data coding to identify themes and gaps will be conducted in Atlas.ti,^79^ a software programme for coding and analysing primarily qualitative data. LS and HT will assist with both the extraction and coding process to ensure consistent coding and boost replicability.

**Table 3:** Extraction tool outline

Category	Extraction fields
Study information	Authors, affiliations, aim, publication date, journal, etc.
Geographic focus	Country, subnational geographic focus, key context notes
Methodology	Aim, design, start date, end date, funding, possible conflicts, limitations
CHW design	Disease(s)/public health focus, population description, role, recruitment, funding, supervision, training, team structure, etc.
Job demands	Content on: workload, work structure, interpersonal conflict, violence and safety
Job resources	Content on: supervision and support, payment and motivation, knowledge and growth, equipment and supplies, trust and teamwork

CHW, community health worker.

The data extraction template was also piloted, with minor changes made to the extraction tool. These changes are reflected above.

#### Data synthesis

Arksey and O’Malley note that a scoping review “does not seek to ‘synthesise’ results, evidence or to aggregate findings…there is no attempt to present a view regarding the ‘weight’ of evidence”.^56^ The authors will explore if there are substantive differences between results in grey versus peer-reviewed literature. Should notable differences be found, results will be displayed in parallel rather than aggregated across these two report types. If a particular report covers more than one country, results will be split into country-specific reports for coding and analysis.

### Patient and public involvement

None.

## Ethics and dissemination

Formal ethical approval is not required, as primary data will not be collected in this study. However, this review is a foundational component of a larger PhD research effort by the lead author, which has received ethical approval (BM25/8/3) from the Biomedical Science Research Ethics Committee at the University of the Western Cape, Cape Town South Africa.

Final results are expected by late 2026, with an overall mapping article submitted for peer review. Consistent with an evidence-mapping approach,^27 28^ data visualisation and tables will be used to make a large volume of information easier to comprehend and interpret.

To address the primary research question, data will be presented in tables organised by job-demand and job-resource themes, enabling the review of similar concepts in parallel. These themes will be informed by the deductive creation of categories for the search strategy as well as inductive analysis of the extracted data. Maps, tables, and narrative descriptions will also be used to answer the secondary questions by exploring the types of research, common and distinct themes within and across countries, and potential knowledge gaps.

Additional articles, reports and conference presentations based on the results may be developed. All dissemination efforts will focus on providing information in a format that can inform the creation or improvement of programmes and will be shared with relevant policymakers, academics and programme implementers engaged within the authors’ networks.

If changes to this protocol are required after its publication, we will provide the amendment date and describe the change or changes, along with their rationale, in any future publications.

## Supplementary material

10.1136/bmjopen-2026-116412 online supplemental file 1

10.1136/bmjopen-2026-116412 online supplemental file 2

10.1136/bmjopen-2026-116412 online supplemental file 3
