# Supplementary material for: Mapping common job demands and job resources for large-scale community health worker programmes in Southern Africa: protocol for a scoping review
Source: BMJ Open. 2026 May 29;16(5):e116412. doi: 10.1136/bmjopen-2026-116412 (PMC13223652; doi:10.1136/bmjopen-2026-116412)
Supplement: online supplemental file 1 [file bmjopen-16-5-s001.pdf]

Supplemental information 1: Piloted PubMed approach and search string

Table 1. Search terms for PubMed piloted on August 12, 2025

**Population**			
**Key Concept**	**Community health workers engaged by the government in PHC programs**		
**Free text terms and natural language terms**	Agentes polivalentes elementares<br>Agentes comunitarios de saude<br>CHW<br>CHWs<br>Community health assistant(*)	Community health worker(*)<br>Enrolled midwife(*)<br>Enrolled nurse(*)<br>Health surveillance assistant(*)<br>Relais<br>RECO	Rural health motivator(*)<br>Village health worker(*)<br>Volunteer CHW(*)<br>Ward-based outreach team(*)<br>Ward-based primary health care outreach team(*)
**MeSH terms**	Community health workers		
**Context**			
**Key Concept**	**Job demands and job resources**		
**Free text terms and natural language terms**	Administration<br>Advancement<br>Aggression<br>Appreciation<br>Attitude<br>Badge<br>Benefits<br>Brochures(*)<br>Bullying(*)<br>Capacity building<br>Career pathway<br>Cohesion<br>Communication<br>Conflict(*)<br>Contracts<br>Core competencies<br>Crime<br>Decision making(*)<br>Delegation<br>Efficiency<br>Emotion (*)<br>Employee(*)<br>Employment<br>Employment(*)<br>Equipment<br>Ethics<br>Feedback	Funding<br>Health records<br>Hiring<br>Incentive<br>Insurance<br>Interpersonal conflict<br>Interpersonal relations<br>Job security<br>Knowledge<br>Management<br>Mandatory programs(*)<br>Medication<br>Motivation<br>Occupational accident<br>Occupational stress(*)<br>Organizational culture<br>Organizational objectives(*)<br>Organizational policy<br>Ownership<br>Pamphlets(*)<br>Plan(*)<br>Political support<br>Pressure<br>Professional development (*)<br>Professional education<br>Programs, mandatory(*)	Promotion(*)<br>Risk management<br>Safety<br>Program development (*)<br>Salary(*)<br>Security<br>Skills development (*)<br>Social environment<br>Stipend<br>Supervision (*)<br>Supplies(*)<br>Task sharing(*)<br>Task shifting(*)<br>Team(*)<br>Time management<br>Training<br>Trauma<br>Uniform<br>Victim<br>Violence<br>Work accident<br>Work culture<br>Work environment<br>Work stress<br>Workforce<br>Workload

**MeSH terms**[1]	Accidents, occupational<br>Attitude<br>Aggression<br>Behavioral symptoms<br>Capacity building<br>Clothing<br>Communication<br>Contracts<br>Crime victims<br>Decision making<br>Decision making, organizational<br>Delegation, professional<br>Education, professional<br>Efficiency<br>Employment<br>Equipment and supplies<br>Financial support	Ethics, professional<br>Feedback<br>Health facility administration<br>Insurance<br>Interpersonal relations<br>Knowledge<br>Management<br>Mandatory programs<br>Models, organization<br>Motivation<br>Occupational health<br>Organizational culture<br>Organizational objectives<br>Organizational policy<br>Ownership<br>Personnel management<br>Planning techniques	Professional practice<br>Professional role<br>Program development<br>Public health administration<br>Public relations<br>Records<br>Resource allocation<br>Risk management<br>Safety<br>Salaries and fringe benefits<br>Security measures<br>Social environment<br>Time management<br>Trauma and stressor related disorders<br>Violence<br>Work<br>Workforce
**Context**			
**Key Concept**	**Southern Africa**		
**Free text terms (Title/Abstract)**	Angola<br>Botswana<br>Comoros<br>Comoro islands<br>Mayotte<br>DRC<br>Democratic republic of the congo<br>Congo-kinshasa<br>DR congo<br>Eswatini<br>Swaziland	Lesotho<br>Basutoland<br>Basotho<br>Madagascar<br>Madagasikara<br>Malagasy<br>Malawi<br>Mauritius<br>Mozambique<br>Mocambique<br>Mocambique<br>Portuguese east africa	Namibia<br>Seychelles<br>Seychellois<br>Seychelloise<br>South africa<br>Tanzania<br>United republic of tanzania<br>Tanganyika<br>Zambia<br>Zimbabwe<br>Rhodesia
**MeSH terms**[2]	Angola<br>Botswana<br>Comoros<br>Democratic Republic of Congo<br>Eswatini<br>Lesotho	Madagascar<br>Malawi<br>Mauritius<br>Mozambique<br>Namibia	Seychelles<br>South Africa<br>Tanzania<br>Zambia<br>Zimbabwe
**Independent search results with filters for date and language**	1,069		

Table 2. Search string for PubMed piloted on August 12, 2025

	Population AND Concept AND Context
**Population**	(“Community health workers”[MeSH Terms] OR “community health worker”[Title/Abstract] OR “enrolled nurse*”[Title/Abstract] OR “enrolled midwi*”[Title/Abstract] OR “health surveillance assistant*”[Title/Abstract] OR “community health assistant*”[Title/Abstract] OR “agentes polivalentes elementares”[Title/Abstract] OR “agentes comunitarios de saude”[Title/Abstract] OR “RECO”[Title/Abstract] OR “rural health motivators”[Title/Abstract] OR “village health worker”[Title/Abstract] OR “relais”[Title/Abstract] OR “volunteer CHWs”[Title/Abstract] OR “CHW”[Title/Abstract] OR “CHWs”[Title/Abstract] OR “Ward-based outreach team”[Title/Abstract] OR “Ward-based primary health care outreach teams”[Title/Abstract])

[1] MeSH terms are not restricted to the Major Topic, subcategories in the MeSH hierarchy are included.

[2] These same terms were also searched for affiliation

**Concept**	(“Efficiency”[MeSH Terms] OR “efficienc*”[Title/Abstract] OR “Time Management”[MeSH Terms] OR “time management”[Title/Abstract] OR “Mandatory Programs”[MeSH Terms] OR “mandatory program”[Title/Abstract] OR “program*, mandatory”[Title/Abstract] OR “pressure”[Title/Abstract] OR “task shar*”[Title/Abstract] OR “emotion*”[Title/Abstract] OR “task shift*”[Title/Abstract] OR “occupational stress*”[Title/Abstract] OR “work stress”[Title/Abstract] OR “workload”[Title/Abstract]) OR (“Work”[MeSH Terms] OR “employment”[Title/Abstract] OR “Employment”[MeSH Terms] OR “Decision making”[MeSH Terms] OR “decision mak*”[Title/Abstract] OR “Decision making, organizational”[MeSH Terms] OR “Models, organizational”[MeSH Terms] OR “Workforce”[MeSH Terms] OR “workforce”[Title/Abstract] OR “Professional Practice”[MeSH Terms] OR “delegation”[Title/Abstract] OR “Delegation, professional”[MeSH Terms] OR “Ethics, professional”[MeSH Terms] OR “ethics”[Title/Abstract] OR “Organizational policy”[MeSH Terms] OR “organizational policy”[Title/Abstract] OR “Professional role”[MeSH Terms] OR “Planning Techniques”[MeSH Terms] OR “plan*”[Title/Abstract] OR “Program Development”[MeSH Terms] OR “Organizational Objectives”[MeSH Terms] OR “organizational objectiv*” OR “program develop*”[Title/Abstract] OR “Ownership”[MeSH Terms] OR “ownership”[Title/Abstract] OR “health records”[Title/Abstract] OR “Records”[MeSH] OR “employ*”[Title/Abstract] OR “job security”[Title/Abstract] OR “hiring”[Title/Abstract]) OR (“Attitude”[MeSH Terms] OR “attitude”[Title/Abstract] OR “Aggression”[MeSH Terms] OR “aggression”[Title/Abstract] OR “Behavioral symptoms”[MeSH Terms] OR “Interpersonal relations”[MeSH Terms] OR “Public Relations”[MeSH Terms] OR “interpersonal conflict”[Title/Abstract] OR “interpersonal relations”[Title/Abstract] OR “bully*”[Title/Abstract] OR “conflict*”[Title/Abstract]) OR (“Accidents, occupational”[MeSH Terms] “work accident”[Title/Abstract] OR “occupational accident”[Title/Abstract] OR “Violence”[MeSH Terms] OR “Trauma and Stressor Related Disorders”[MeSH Terms] OR “violence”[Title/Abstract] OR “safety”[Title/Abstract] OR “Safety”[MeSH Terms] OR “Crime victims”[MeSH Terms] OR “Occupational health”[MeSH Terms] OR “Risk Management”[MeSH Terms] OR “Security Measures”[MeSH Terms] OR “risk management”[Title/Abstract] OR “security”[Title/Abstract] OR “crime”[Title/Abstract] OR “trauma*”[Title/Abstract] OR “victim”[Title/Abstract]) OR (“Feedback”[MeSH Terms] OR “feedback”[Title/Abstract] OR “Personnel Management”[MeSH Terms] OR “management”[Title/Abstract] OR “Public Health Administration”[MeSH Terms] OR “Health Facility Administration”[MeSH Terms] OR “administration”[Title/Abstract] OR “appreciation”[Title/Abstract] OR “political support”[Title/Abstract] OR “supervis*”[Title/Abstract]) OR (“Salaries and fringe benefits”[MeSH Terms] OR “salar*”[Title/Abstract] OR “benefits”[Title/Abstract] OR “Motivation”[MeSH Terms] OR “motivation”[Title/Abstract] OR “Contracts”[MeSH Terms] OR “contracts”[Title/Abstract] OR “Insurance”[MeSH Terms] OR “insurance”[Title/Abstract] OR “stipend”[Title/Abstract] OR “financial support”[Title/Abstract] OR “incentive”[Title/Abstract] OR “funding”[Title/Abstract]) OR (“Education, professional”[MeSH Terms] OR “Knowledge”[MeSH Terms] OR “Capacity building”[MeSH Terms] OR “career pathway”[Title/Abstract] OR “professional devel*”[Title/Abstract] OR “professional education”[Title/Abstract] OR “knowledge”[Title/Abstract] OR “capacity building”[Title/Abstract] OR “core competencies”[Title/Abstract] OR “Advancement”[Title/Abstract] OR “promot*”[Title/Abstract] OR “Skill* devel*”[Title/Abstract] OR “training”[Title/Abstract]) OR (“Equipment and Supplies”[MeSh Terms] OR “Resource allocation”[MeSH Terms] OR “Clothing”[MeSH Terms] OR “uniform”[Title/Abstract] OR “badge”[Title/Abstract] OR “equipment”[Title/Abstract] OR “suppl*”[Title/Abstract] OR “medication”[Title/Abstract] OR “brochure*”[Title/Abstract] OR “pamphlet*”[Title/Abstract]) OR (“Social environment”[MeSH Terms] OR “work environment”[Title/Abstract] OR “social environment”[Title/Abstract] OR “Communication”[MeSH Terms] OR “communication”[Title/Abstract] OR “Organizational Culture”[MeSH Terms] OR “work culture”[Title/Abstract] OR “organizational culture”[Title/Abstract] OR “team*”[Title/Abstract] OR “cohesion”[Title/Abstract])
**Context**	("Angola"[MeSH Terms] OR "angola"[Transliterated Title] OR "angola"[Affiliation]) OR ("Botswana"[MeSH Terms] OR "botswana"[Title/Abstract] OR "botswana"[Affiliation] OR "botswana"[Transliterated Title]) OR ("Democratic Republic of the Congo"[MeSH Terms] OR ("Comoros"[MeSH Terms] OR “Comoro Islands”[Title/Abstract] OR “Mayotte”[Title/Abstract] OR "comoros"[Affiliation]) OR "Democratic Republic of the Congo"[Title/Abstract] OR "Democratic Republic of the Congo"[Affiliation] OR "Congo-Kinshasa"[Title/Abstract] OR "Congo-Kinshasa"[Affiliation] OR "DR Congo"[Title/Abstract] OR "DR Congo"[Affiliation] OR "Zaire"[Affiliation]) OR ("Eswatini"[MeSH Terms] OR "eswatini"[Title/Abstract] OR "Eswatini"[Transliterated Title] OR "swaziland"[Title/Abstract] OR "Swaziland"[Transliterated Title] OR "Eswatini"[Affiliation] OR "Swaziland"[Affiliation] OR "swazi"[Title/Abstract]) OR ("Lesotho"[MeSH Terms] OR "lesotho"[Title/Abstract] OR "Lesotho"[Transliterated Title] OR "Lesotho"[Affiliation] OR "Basutoland"[Title/Abstract] OR "Basutoland"[Transliterated Title] OR "Basotho"[Title/Abstract]) OR ("Madagascar"[MeSH Terms] OR "Madagascar"[Title/Abstract] OR "Madagascar"[Transliterated Title] OR "Madagascar"[Affiliation] OR "Madagasikara"[All Fields] OR "Malagasy"[Title/Abstract] OR "madagascan*"[Title/Abstract]) OR ("Malawi"[MeSH Terms] OR "malawi"[Title/Abstract] OR "Malawi"[Transliterated Title] OR "Malawi"[Affiliation]) OR ("Mauritius"[MeSH Terms] OR "Mauritius"[Title/Abstract] OR "Mauritius"[Affiliation] OR "Mauritian"[Title/Abstract]) OR ("Mozambique"[MeSH Terms] OR "mozambique*"[Title/Abstract] OR "Mozambique"[Transliterated Title] OR "Mozambique"[Affiliation] OR "Mocambique"[Affiliation] OR "mozambique"[Title/Abstract] OR "Mocambique"[Transliterated Title] OR "Mocambique"[Title/Abstract] OR "portuguese east africa"[Title/Abstract]) OR ("Namibia"[MeSH Terms]

	OR "namibia"[Title/Abstract] OR "Namibia"[Transliterated Title] OR "Namibia"[Affiliation]) OR (“Seychelles”[MeSH Terms] OR "Seychelles"[Title/Abstract] OR "Seychelles"[Transliterated Title] OR "Seychelles"[Affiliation] OR "Seychellois"[Title/Abstract] OR "Seychelloise"[Title/Abstract]) OR ("South Africa"[MeSH Terms] OR "South Africa"[Title/Abstract] OR "South Africa"[Transliterated Title] OR "South Africa"[Affiliation]) OR ("Tanzania"[MeSH Terms] OR "tanzania"[Affiliation] OR "tanzania"[Transliterated Title] OR “United Republic of Tanzania”[Title/Abstract] "Tanganyika"[Title/Abstract]) OR ("Zambia"[MeSH Terms] OR "zambia"[Title/Abstract] OR "Zambia"[Transliterated Title] OR "Zambia"[Affiliation]) OR ("Zimbabwe"[MeSH Terms] OR "zimbabwe"[Title/Abstract] OR "Zimbabwe"[Transliterated Title] OR "Zimbabwe"[Affiliation] OR "Rhodesia"[Title/Abstract] OR "Rhodesia"[Transliterated Title] OR "Rhodesia"[Affiliation] OR "rhodesia"[Title/Abstract])
**Filters**	2008 to 2025, English
**Pilot results**	1,069
