# Supplementary figures and images for: Mapping common job demands and job resources for large-scale community health worker programmes in Southern Africa: protocol for a scoping review
Source: BMJ Open. 2026 May 29;16(5):e116412. doi: 10.1136/bmjopen-2026-116412 (PMC13223652; doi:10.1136/bmjopen-2026-116412)

## Supplemental information 2: Decision algorithm for title/abstract screening

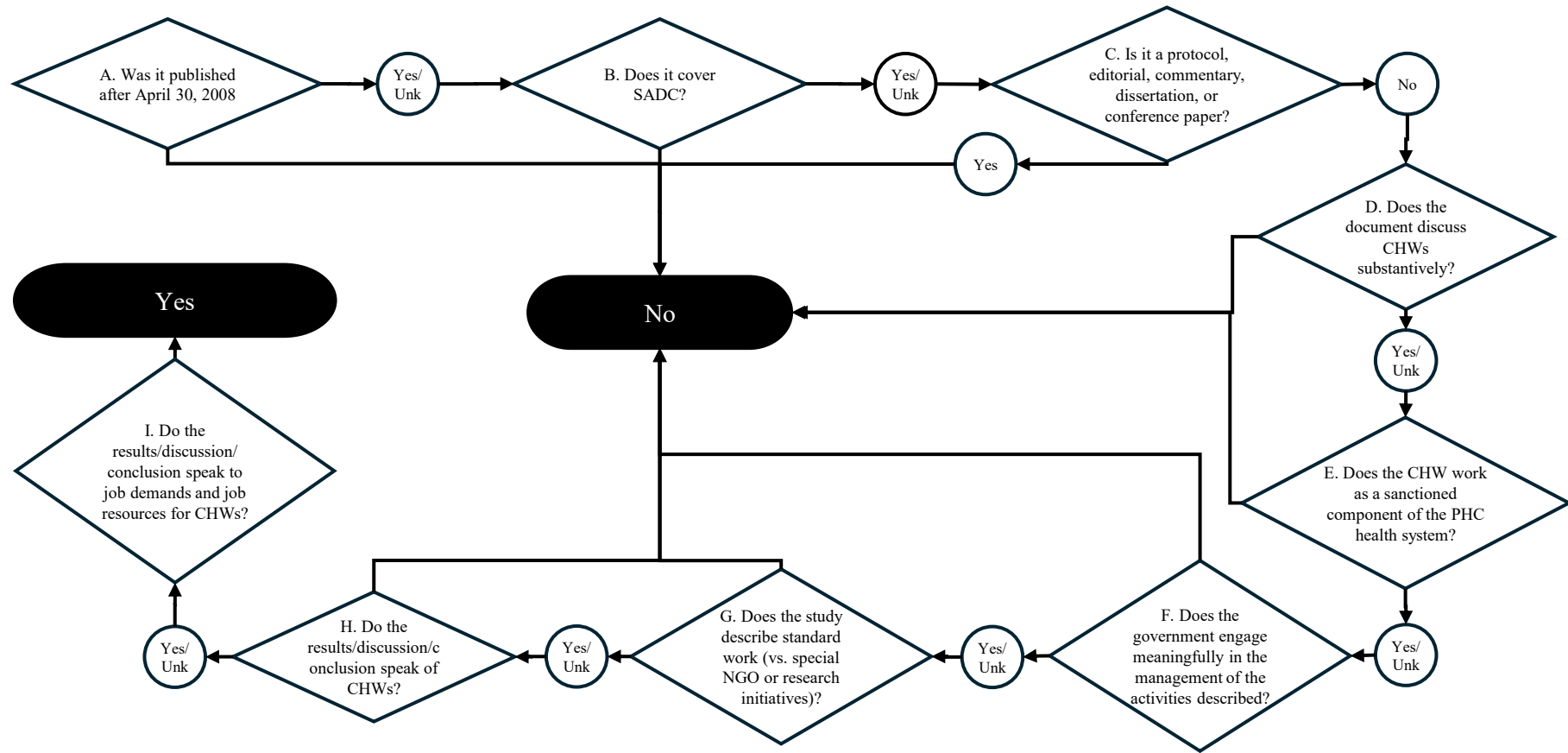

Supplement: online supplemental file 2 [file bmjopen-16-5-s002.pdf]

# Supplemental information 3: Decision algorithm for full-text review

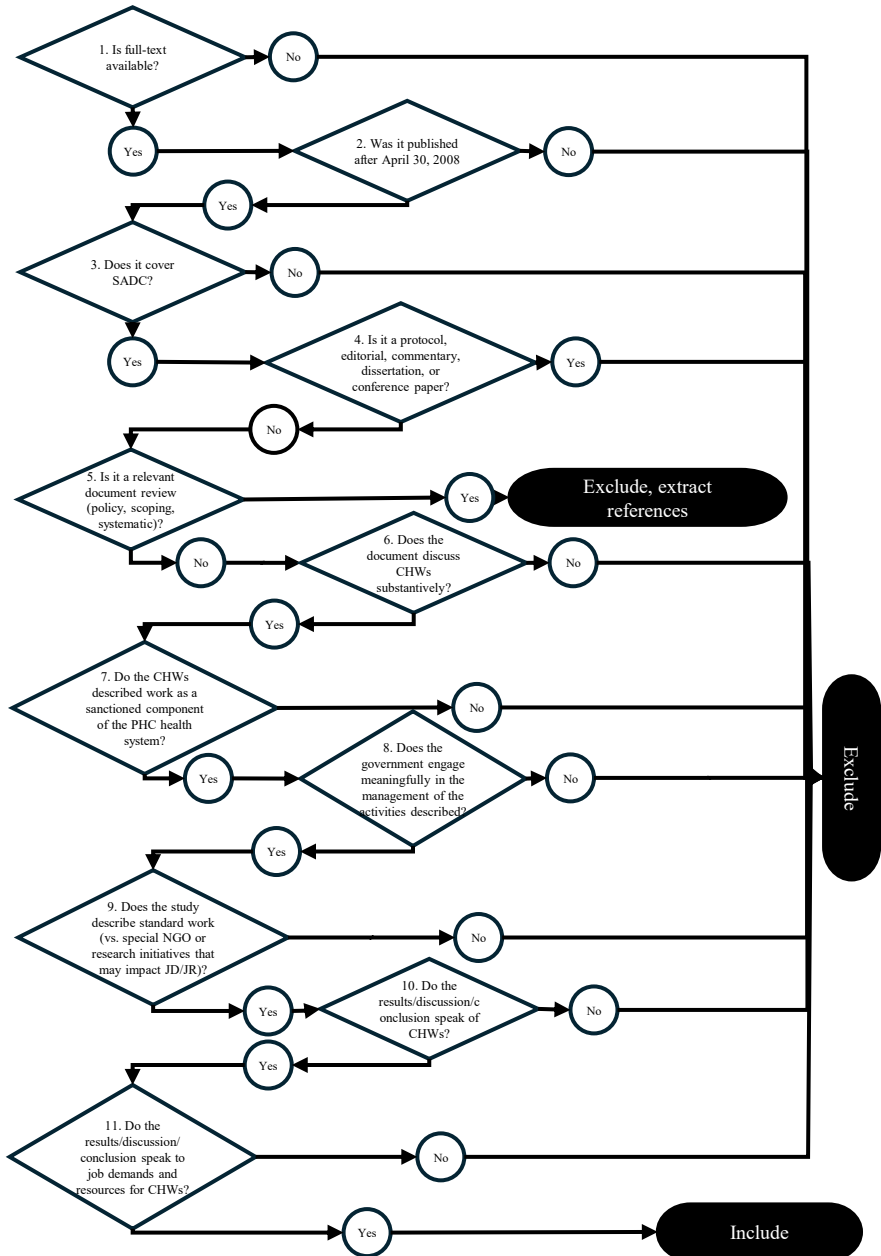

Supplement: online supplemental file 3 [file bmjopen-16-5-s003.pdf]
