# Supplementary material for: Mapping common job demands and job resources for large-scale community health worker programmes in Southern Africa: protocol for a scoping review
Source: BMJ Open. 2026 May 29;16(5):e116412. doi: 10.1136/bmjopen-2026-116412 (PMC13223652; doi:10.1136/bmjopen-2026-116412)
Supplement: Supplementary file 4 [file bmjopen-2026-116412.reviewer_comments.pdf]

PEER REVIEW HISTORY

BMJ Open publishes all reviews undertaken for accepted manuscripts. Reviewers are asked to complete a checklist review form and are provided with free text boxes to elaborate on their assessment. These free text comments are reproduced below.

ARTICLE DETAILS

Title (Provisional)

Mapping common job demands and job resources for large-scale community health worker programs in Southern Africa: protocol for a scoping review

Authors

Coley, Rachel; Shuro, Linda; Coetzee, Renier; Tabana, Hanani

VERSION 1 - REVIEW

**Reviewer**	**1**
**Name**	**Steenbeek, Audrey**
**Affiliation**	**Dalhousie University, nursing**
**Date**	**08-Mar-2026**
**COI**	**None**

This protocol addresses an important topic and has the potential to contribute meaningfully to the literature on community health worker programs. However, the manuscript would benefit from clearer organization, improved methodological transparency, and more consistent use of sub-headings. Addressing these issues will significantly strengthen the manuscript and improve its readability

Thank you for the opportunity to review this manuscript. Overall, this is a worthwhile and timely endeavour. Scoping reviews are particularly valuable for mapping existing evidence and laying the groundwork for future research, especially in the context of community health worker (CHW) programs. The topic is important and relevant for strengthening health systems in Southern Africa.

However, I found the manuscript difficult to follow due to limited structural organization and insufficient use of sub-headings. Improving the structure would greatly enhance readability and replicability. In particular, the methods section requires clearer organization to ensure that readers can easily understand and reproduce the review process.

Overall Structural Suggestions

I recommend reorganizing the manuscript to improve clarity and methodological transparency. Specifically:

- Begin the methods section with clearly defined review questions. The current statements appear to be objectives rather than research questions.
- In the Methods and Analysis section, consider the following structure:
    - Eligibility Criteria
        - Participants
        - Concept
        - Context
    - Types of Literature
    - Information Sources
    - Search Strategy
    - Study Records
        - Data Management
        - Selection Process
        - Data Collection
        - Data Synthesis

Additionally, the manuscript would benefit from including a Patient and Public Involvement (PPI) statement, as this is commonly expected in BMJ open.

Specific Comments

Abstract

- Under Methods and Analysis, rather than stating that the Population–Concept–Context (PCC) framework guided the search strategy, the authors should explicitly describe the population, concept, and context within the abstract.
- The rationale for restricting the search to April 30, 2008 onwards should be clarified. It may be more appropriate to search from database inception to a specified end date to ensure comprehensive coverage of the literature.
- The abstract should also indicate when the scoping review will commence, as ideally the review should begin after the protocol is published.
- Consider including additional databases to improve comprehensiveness, such as:
    - MEDLINE (Ovid)
    - Embase (Elsevier)
    - PsycINFO (EBSCO)
    - Academic Search Premier (EBSCO)
    - Business Source Complete (EBSCO)
    - ProQuest Dissertations and Theses Global

- Under Strengths and Limitations, the language referring to a "systematic approach" should be reconsidered, as this study is a scoping review rather than a systematic review.

Introduction

- Provide a clearer definition of community health workers (CHWs) early in the introduction.
- The phrase "vertical, disease-specific CHW models" should be explained or illustrated with an example. It would also be helpful to clarify why these programs are considered "large-scale." (Introduction, line 9).
- At line 18, challenges related to governance, management, and funding are mentioned. These should be described in greater detail, as they form a key part of the problem being addressed.
- At line 23, the manuscript contains numerous acronyms, which makes the text difficult to read. Consider reducing the number of acronyms or ensuring they are clearly explained.
- Lines 27–33 appear somewhat out of place in the introduction and would likely be better positioned in the methods section.
- Lines 36–41 could be improved by first explaining what scoping reviews are best suited for and then linking this rationale to the current study.
- At line 46, the manuscript references PRISMA. The correct guideline is Preferred Reporting Items for Systematic Reviews and Meta-Analyses Extension for Scoping Reviews (PRISMA-ScR). Additionally, PRISMA-ScR is used for reporting, not for protocol development. Arksey and O'Malley's framework is more appropriate for informing the development of a scoping review protocol.

Methods

Review Questions

(Page 6, line 22)

The statements presented are objectives rather than research questions. The authors should clearly articulate a primary research question, and if relevant, sub-questions.

Conceptual Framework

(Page 7)

The section beginning on page 7, line 3 introduces the Job Demands–Resources (JD-R) model, but it lacks a clear sub-heading. I recommend adding a Conceptual Framework sub-section.

The content currently in page 5, lines 27–33 could also be moved here to improve conceptual flow.

Organization of the Methods Section

The following sections would benefit from clearer structure and sub-headings:

- Page 7 (lines 28–55) and page 8 (lines 4–5) require improved organization. Some content may belong in the introduction, while other sections should be organized under headings such as Concept and Context.
- Page 8 (lines 15–22): Please clarify how Hodgins' taxonomy and Schaufeli and Taris's list of job demands and resources will be integrated with the JD-R model.
- Page 9 (lines 41–49) should be organized under clearer headings such as Concept and Context.

Information Sources

- Page 9 (line 51) to page 10 (line 10) should be presented under a sub-heading such as Information Sources.

Types of Literature

- Page 10 (lines 12–27) would be better placed under a heading such as Types of Literature.
- The justification for starting the search on April 30, 2008 should be reconsidered. It may be preferable to search from database inception to a defined end date to avoid missing relevant earlier studies.

Data Management

- Lines 41–42 should include an explanation of Papers by ReadCube, as not all readers may be familiar with this software.

Data Charting

- In Stage 4 (Charting the Data), the authors should clarify how grey literature will be extracted and charted.

Search Strategy

- Table 4 should include the complete search strategy, including all keywords, Boolean operators, and database-specific terms, to ensure transparency and reproducibility.

Reviewer **2**

Name **Gershuni, Olga**

Affiliation **Department of International Health, School CAPHRI, Faculty of Health, Medicine and Life Sciences, Maastricht University, Maastricht, The Netherlands.**

Date **01-Apr-2026**

COI **None**

Thank you for submitting the review protocol. This is a needed study that focuses on the well-being of community health workers in Southern Africa. I agree that the issues you raised are important and need attention, especially given the issues in the communities.

Several aspects need clarification or strengthening before proceeding with your work.

The focus on the job demands-resources model is important; however, why is it the most preferred model for investigating community health workers' well-being?

Although you mention that the tasks and responsibilities are not the areas for your research, it is important to describe who those professionals are and how the contexts (and health systems) within Southern African countries can be comparable. Can the recommendations and conclusions be applied to all countries in this region? Who will be the receiving stakeholders?

Also, you mention the work in large-scale CHW programs; this is interesting because it apparently also leads to high levels of associated stress related to performing daily duties. Lines 36-41 are thus not entirely clear as they do not entirely match what you are saying in lines 25-27.

The initial search strategy in scientific databases is described. How are you planning to carry out the grey literature searches? How will the information be blended? You mention narrative synthesis; could you clarify how the analysis will be carried out?

Please include the dates/timeline for the study you plan to carry out.

VERSION 1 - AUTHOR RESPONSE

Reviewer 1 Comments

- I found the manuscript difficult to follow due to limited structural organization and insufficient use of sub-headings. Improving the structure would greatly enhance readability and replicability. In particular, the methods section requires clearer organization to ensure that readers can easily understand and reproduce the review process. *This feedback is appreciated and addressed throughout the manuscript as noted in tracked changes and alongside the specific comments below.*

- Overall Structural Suggestions: I recommend reorganizing the manuscript to improve clarity and methodological transparency. Specifically:

  Begin the methods section with clearly defined review questions. The current statements appear to be objectives rather than research questions.

  In the Methods and Analysis section, consider the following structure:

  Eligibility Criteria: Participants, Concept, Context

  Types of Literature

  Information Sources

  Search Strategy

  Study Records: data management, selection process, data collection, data synthesis

  Significant reorganization to the text has been done to provide clarity and flow. The majority of the headings above are utilized, with some additional and/or more specific headings used to provide additional clarity.

- Additionally, the manuscript would benefit from including a Patient and Public Involvement (PPI) statement, as this is commonly expected in BMJ open. *This is now included, noted in tracked changes and flagged with a comment in the marked version.*

- Abstract: Under Methods and Analysis, rather than stating that the Population–Concept–Context (PCC) framework guided the search strategy, the authors should explicitly describe the population, concept, and context within the abstract. *This has been added.*

- Abstract: The rationale for restricting the search to April 30, 2008 onwards should be clarified. It may be more appropriate to search from database inception to a specified end date to ensure comprehensive coverage of the literature. *This has been briefly clarified in the abstract. As this date ushered in a resurgence of large-scale CHW programs for the relevant context, earlier searches would not identify relevant content. The pilot did not identify any relevant studies published before 2011 as it took time to establish programs and publish literature on them.*

- Abstract: The abstract should also indicate when the scoping review will commence, as ideally the review should begin after the protocol is published. *This has been added.*

- Abstract: Consider including additional databases to improve comprehensiveness, such as: MEDLINE (Ovid), Embase (Elsevier), PsycINFO (EBSCO), Academic Search Premier (EBSCO), Business Source Complete (EBSCO), ProQuest Dissertations and Theses Global. *This suggestion is appreciated, however the addition of new database searches suggested is beyond our current resource limitations given its likely limited impact on the findings of this study. MEDLINE is already covered under the PubMed search. Business Source Complete focuses primarily on key business areas such as accounting, finance, management, and marketing. While management has some overlap with the topic area, the focus on health-related journals will cover most articles published in health management-focused journals, of which there are many. The suggestion of ProQuest does not align with the inclusion criteria, as dissertations and theses are excluded. Searching Embase would identify most of the items already found via the PubMed search (and therefore deduplicated), as well as additional content on drug or pharmacy topics, however those are unlikely to be relevant to the topic at hand. Embase is also known for broader coverage of European journals, though again, as the*

focus of the research is on Southern Africa, this is not considered a major advantage. The initial search pilot of five databases included within the article identified 5,242 studies and further work to deduplicate those findings have led to more than 3,000 studies for review. The additional use of a purposive citating mining approach and the inclusion of a cross-cutting, topical database (CHW Central), further enhance the authors' confidence that the majority of relevant evidence will be identified with the proposed approach. However, as is true with all scoping reviews, some relevant literature will be missed. This limitation will be noted in any manuscript resulting from this review.

- Abstract: Under Strengths and Limitations, the language referring to a "systematic approach" should be reconsidered, as this study is a scoping review rather than a systematic review. *This has been adjusted.*

- Introduction: Provide a clearer definition of community health workers (CHWs) early in the introduction. *A brief introductory definition has been included in the first paragraph, with a more formal definition relevant to the eligibility criteria of this study included under Eligibility Criteria.*

- Introduction: The phrase "vertical, disease-specific CHW models" should be explained or illustrated with an example. It would also be helpful to clarify why these programs are considered "large-scale." (Introduction, line 9). *This section has been reworked to provide more clarity.*

- Introduction: At line 18, challenges related to governance, management, and funding are mentioned. These should be described in greater detail, as they form a key part of the problem being addressed. *This topic has been fleshed out within the introduction.*

- Introduction: At line 23, the manuscript contains numerous acronyms, which makes the text difficult to read. Consider reducing the number of acronyms or ensuring they are clearly explained. *Universal health coverage (UHC) is now spelled out here and in the other two*

mentions in the manuscript. Sustainable Development Goals (SDGs) is now spelled out here and in the other two mentions in the manuscript as well.

- Introduction: Lines 27–33 appear somewhat out of place in the introduction and would likely be better positioned in the methods section. *This has been adjusted.*
- Introduction: Lines 36–41 could be improved by first explaining what scoping reviews are best suited for and then linking this rationale to the current study. *This has been clarified in the relevant paragraph.*
- Introduction: At line 46, the manuscript references PRISMA. The correct guideline is Preferred Reporting Items for Systematic Reviews and Meta-Analyses Extension for Scoping Reviews (PRISMA-ScR). Additionally, PRISMA-ScR is used for reporting, not for protocol development. Arksey and O'Malley's framework is more appropriate for informing the development of a scoping review protocol. *This has been adjusted in light of this comment and one from the Editor.*
- Methods: Review Questions (Page 6, line 22) The statements presented are objectives rather than research questions. The authors should clearly articulate a primary research question, and if relevant, sub-questions. *This has been adjusted based on the reviewer's suggestion.*

- Methods: Conceptual Framework (Page 7): The section beginning on page 7, line 3 introduces the Job Demands–Resources (JD-R) model, but it lacks a clear sub-heading. I recommend adding a Conceptual Framework sub-section. *A subheading has been added. To prevent confusion with the PCC Framework, the sub-section is entitled the Job Demands-Resources Model and is now found at the end of the introduction as its content should be described before the research questions, which are based on the framework, are articulated.*

- Methods: The content currently in page 5, lines 27–33 could also be moved here to improve conceptual flow. *The location of this content has been updated.*

- Organization of the Methods Section: The following sections would benefit from clearer structure and sub-headings: Page 7 (lines 28–55) and page 8 (lines 4–5) require improved organization. Some content may belong in the introduction, while other sections should be organized under headings such as Concept and Context. *The content has been reorganized.*

- Page 9 (lines 41–49) should be organized under clearer headings such as Concept and Context. Information Sources. Page 9 (line 51) to page 10 (line 10) should be presented under a sub-heading such as Information Sources. *The content has been reorganized.*

- Types of Literature: Page 10 (lines 12–27) would be better placed under a heading such as Types of Literature. *The content has been reorganized.*

- Methods: Page 8 (lines 15–22): Please clarify how Hodgins' taxonomy and Schaufeli and Taris's list of job demands and resources will be integrated with the JD-R model. *This is now articulated within Table 1 to enhance clarity and reduce wordiness.*

- Methods: The justification for starting the search on April 30, 2008 should be reconsidered. It may be preferable to search from database inception to a defined end date to avoid missing relevant earlier studies. *Due to the nature of these programs, searching before 2008 is unlikely to identify content relevant to the research questions, as large-scale programs were not widely implemented prior to the Declaration of Ouagadougou. As noted on an earlier comment, the pilot actually did not identify any articles for inclusion prior to a 2011 publication date.*

- Data Management: Lines 41–42 should include an explanation of Papers by ReadCube, as not all readers may be familiar with this software. *A clause has been added to note it is a reference management software system. To ensure consistency, a similar clause has been added for Covidence and for Atlas.ti, which has been added into the Charting the Data section.*

- Data Charting: In Stage 4 (Charting the Data), the authors should clarify how grey literature will be extracted and charted. *This has been articulated with the clarification that all grey literature and peer-reviewed literature will follow a similar extraction approach.*
- Search Strategy: Table 4 should include the complete search strategy, including all keywords, Boolean operators, and database-specific terms, to ensure transparency and reproducibility. *Table 4 describes the results of the pilot, not the search strategy (that is summarized in Table 2. The complete PubMed search strategy is included in Supplemental Information 1, including all MeSH and free text terminology and the Boolean operators. This use of a single search strategy aligns with recent BMJ Open Scoping Review Protocols including Lee et al's Mapping scoping reviews in neurosurgery: a scoping review protocol, Khalil et al.'s "Treatment options for chronic hypotony: a scoping review protocol, and Hibusu, Mianda and Akintola's School health initiatives in low-income and middle-income countries: a scoping review protocol.*

  All search strategies are available and could be included in this Supplement if preferred by the journal.

Reviewer 2 Comments

- The focus on the job demands-resources model is important; however, why is it the most preferred model for investigating community health workers' well-being? *A new subheading for the JD-R model has been added, including two paragraphs that explain why this model is the most relevant for this activity.*
- Although you mention that the tasks and responsibilities are not the areas for your research, it is important to describe who those professionals are and how the contexts (and health systems) within Southern African countries can be comparable. *Additional information into the role and issues related with these programs has been added to the introduction. The Context section under eligibility criteria also provides detail on why it is appropriate to compare SADC countries and their CHW programs. Additional information on the receiving stakeholders has been added to the final section.*

- Can the recommendations and conclusions be applied to all countries in this region? *While this research may identify cross-cutting findings for all countries, results will primarily be analysed at the country level. This is now more clearly explained in the data synthesis section. Recommendations will be limited and framed according to the nature of the evidence that is found (where, what type of program, etc.).*
- Who will be the receiving stakeholders? *Additional clarity on this has been added to the final section.*
- Also, you mention the work in large-scale CHW programs; this is interesting because it apparently also leads to high levels of associated stress related to performing daily duties. Lines 36-41 are thus not entirely clear as they do not entirely match what you are saying in lines 25-27. *Content has been reorganized and new content added to try and address this issue.*
- The initial search strategy in scientific databases is described. How are you planning to carry out the grey literature searches? How will the information be blended? You mention narrative synthesis; could you clarify how the analysis will be carried out? *This has been clarified at several points in the methods section including search strategy and data synthesis.*
- Please include the dates/timeline for the study you plan to carry out. *Two timeline clarifications have been added. Without knowing when the protocol will be accepted and published, additional specificity is challenging.*

VERSION 2 - REVIEW

Reviewer 2

Name Gershuni, Olga

Affiliation Department of International Health, School CAPHRI, Faculty of Health, Medicine and Life Sciences, Maastricht University, Maastricht, The Netherlands.

Date 13-May-2026

COI

It was good to read the adjusted protocol.
